# Hydrogel-Based Vitreous Substitutes

**DOI:** 10.3390/ijms26178406

**Published:** 2025-08-29

**Authors:** Soheil Sojdeh, Amirhosein Panjipour, Zahra Bibak Bejandi, Majid Salehi, Amal Yaghmour, Zohreh Arabpour, Ali R. Djalilian, R. V. Paul Chan

**Affiliations:** 1 Department of Ophthalmology and Visual Science, University of Illinois, Chicago, IL 60612, USA; sojdesoheil@gmail.com (S.S.); rvpchan@uic.edu (R.V.P.C.); 2 Department of Tissue Engineering, School of Medicine, Shahroud University of Medical Sciences, Shahroud 3614773955, Iran

**Keywords:** crosslinking strategies, vitreous substitutes, polymeric hydrogel

## Abstract

Hydrogel-based vitreous substitutes have been considered as a potential solution for the treatment of retinal disorders, especially when the natural vitreous body is damaged due to trauma, disease, or surgery. With their high-water content, biocompatibility, and tunable mechanical properties, these hydrogels offer a promising alternative to traditional vitreous substitutes. This review explores the role of polymers and crosslinkers in the development of hydrogel-based substitutes, focusing on how these components contribute to the structure and function of hydrogels. The choice of natural polymers, such as hyaluronic acid and collagen, or synthetic ones, such as polyethylene glycol and polyvinyl alcohol, is crucial to mimic the transparency and flexibility of the vitreous body. Crosslinking methods, including physical, chemical, and enzymatic approaches, help control the gelation process and enhance the mechanical strength of the hydrogel. Furthermore, this review demonstrates how these hydrogels interact with biological tissues, which enhances biocompatibility, cell growth, and tissue repair. This review also discusses the challenges and future directions in improving these hydrogels, particularly in terms of long-term stability, integration with ocular tissues, and appropriate mechanical properties. Overall, hydrogel-based vitreous substitutes have significant potential to improve surgical outcomes and restore vision for patients with vitreous injury.

## 1. Introduction

Vitrectomy is a widely used surgical technique for treating retinal detachment and other serious eye conditions. It involves the removal of the vitreous humor, a clear, gel-like substance that fills the space between the lens and the retina in the back of the eye. This gelatinous matrix is made up of 98–99% water stabilized by a complex matrix of collagen (type II, VXI, and IX), hyaluronic acid, proteoglycans, soluble proteins, metabolites, and ions, giving it the viscoelasticity and transparency essential for maintaining normal vision [1,2]. Once removed during surgery, however, the vitreous body cannot regenerate on its own, making it necessary to use a substitute to preserve the shape of the eye, support the retina, and maintain intraocular pressure [3]. For decades, clinicians have relied on traditional vitreous substitutes such as expansile gases (like sulfur hexafluoride and perfluoro propane), balanced salt solutions, and silicone oils [4]. While these materials can be effective in the short term, especially in helping to reattach the retina, they are far from ideal. Many patients experience complications like cataracts, increased intraocular pressure, inflammation, or even emulsification of the substitute over time, making these materials unsuitable for long-term use [5]. In response to these challenges, researchers have turned their attention to hydrogel-based vitreous substitutes, which offer several promising advantages. Thanks to their high-water content, tunable mechanical properties, and excellent biocompatibility, hydrogels are better suited to mimicking the native vitreous environment [6]. Importantly, they can also be engineered to gel in situ, meaning they can be injected into a liquid and solidify inside the eye, allowing for minimally invasive application and a more natural conformation to the eye’s internal structure [7]. Table 1 provides a comparative overview of current and emerging vitreous substitutes, highlighting their physical properties, clinical benefits, limitations, and relevant applications. It also includes key references to support the scientific basis and development status of each material class, from traditional tamponade agents to advanced hydrogel-based systems.

This review takes a closer look at how different polymers and crosslinking strategies contribute to the development and performance of hydrogel-based vitreous substitutes. Natural polymers like hyaluronic acid and collagen are particularly attractive due to their transparency, degradability, and excellent compatibility with eye tissue. On the other hand, synthetic polymers such as polyethylene glycol and polyvinyl alcohol offer greater mechanical strength and allow for more precise control over degradation and gelation behaviors [5]. The way these polymers are crosslinked, whether through physical interactions, chemical bonding, or enzymatic reactions, also plays a crucial role in determining the final properties of the hydrogel, such as its strength, stability, and gelation time [5,8]. Beyond mechanical and optical properties, one of the most critical factors in designing these hydrogels is how they interact with the biological environment of the eye. Ideal vitreous substitutes must not only be safe and non-toxic but also integrate smoothly with surrounding tissues to avoid triggering inflammation or immune responses. They should facilitate nutrient transport and support retinal function without disrupting the delicate ocular balance [9]. Additionally, the injectability of the synthetic hydrogels as vitreous substitutes inside the eye offers significant practical advantages for surgeons, making these materials even more attractive for clinical use [10].

**Table 1:** Comparative overview of vitreous substitutes.

Type	Examples	Advantages	Limitations	Applications	Ref.
Gases	SF_6_, C_3_F_8_	- Tamponade effect - Resorbs spontaneously - Easy injection	- Nontransparent during expansion - No long-term support - Risk of IOP rise	Retinal detachment, macular hole surgery	[11,12,13]
Silicone Oils	1000 cSt, 5000 cSt silicone oil	- Long-term tamponade - Biocompatible - Transparent	- Requires surgical removal - Risk of emulsification - IOP elevation	Complicated retinal detachment, proliferative vitreoretinopathy	[14,15,16]
Heavy Silicone Oils	Densiron-68, Oxane HD	- Settles inferiorly - Good for inferior pathology	- Heavier than water - Higher risk of inflammation and emulsification	Inferior retinal detachment	[17]
Perfluorocarbon Liquids (PFCLs)	PFO, perfluorodecalin	- High specific gravity - Effective in flattening retina	- Not suitable for long-term use - Retinal toxicity if retained	Retinal reattachment	[18,19,20]
Hydrogels (Natural)	Hyaluronic-acid-based, collagen	- ECM-mimicking - Biodegradable - Biocompatible	- Rapid degradation - Weak mechanical properties	Experimental vitreous substitute	[21]
Hydrogels (Synthetic)	PEG-based, PVA, polyacrylamide	- Tunable properties - Controlled degradation - Injectable	- Requires crosslinking - Possible cytotoxicity from residues	Vitreous replacement in preclinical studies	[8,22]
Smart Hydrogels	Thermo-responsive, pH-sensitive	- In situ gelling - Controlled drug delivery - Responsive to stimuli	- Complex formulation - Regulatory hurdles	Targeted drug delivery, long-acting tamponade	[23,24]
Hydrophilic Polymers	PVA, polyHEMA	- Highwater content, optical clarity, biocompatibility	- Poor long-term stability, limited tamponade effect	Vitreous substitute	[25]

## 2. Types of Polymers

**Figure 1:**
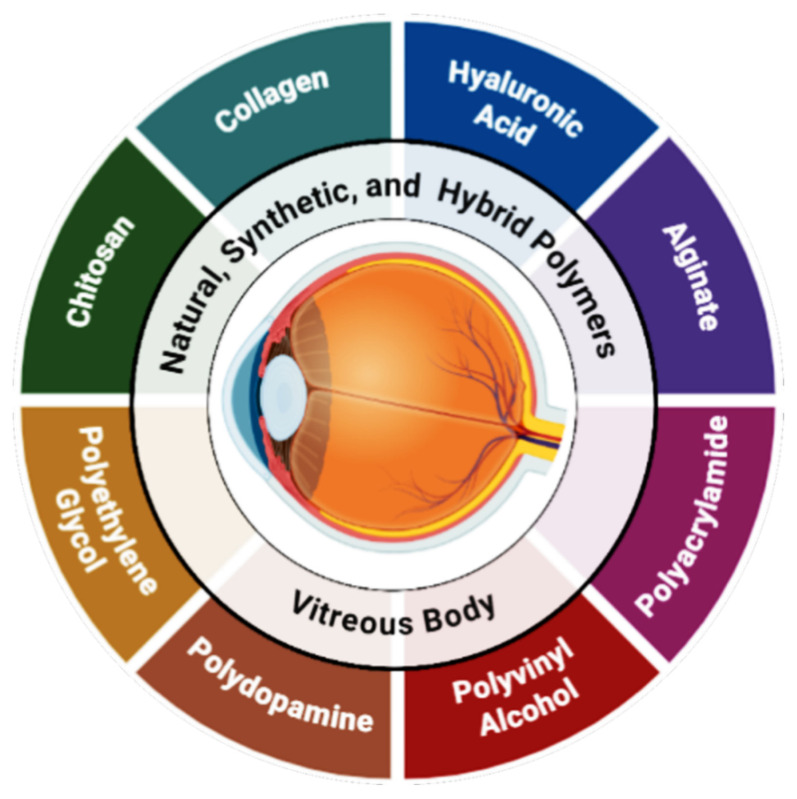
Suitable polymers for the fabrication of injectable hydrogel-based vitreous substitutes.

Biomedical uses of natural and synthetic polymers, especially vitreous replacements for ophthalmology, are promising. The best vitreous replacement should match native vitreous’s clarity, viscoelasticity, and biocompatibility while remaining stable in the eye. Figure 1 shows that polymers utilized for this purpose include natural, synthetic, and hybrid, each having pros and cons.

### 2.1. Natural Polymers

Hyaluronic acid and collagen are natural polymers that are good candidates for testing as vitreous replacements since they are the primary parts of the original vitreous. They have been considered to be the best candidates for making new vitreous replacements because they are hydrophilic compounds that work better with the body than the present hydrophobic synthetic tamponade materials [26]. Native vitreous contains non-immunogenic hyaluronic acid, which is significant for generating natural vitreous replacements due to its optical and mechanical properties, utility, and biocompatibility. Schulz et al. [27]. examined the safety and efficacy of hyaluronic-acid-based vitreous replacements in 21 bulbi eyes in a retrospective interventional investigation.

**Figure 2:**
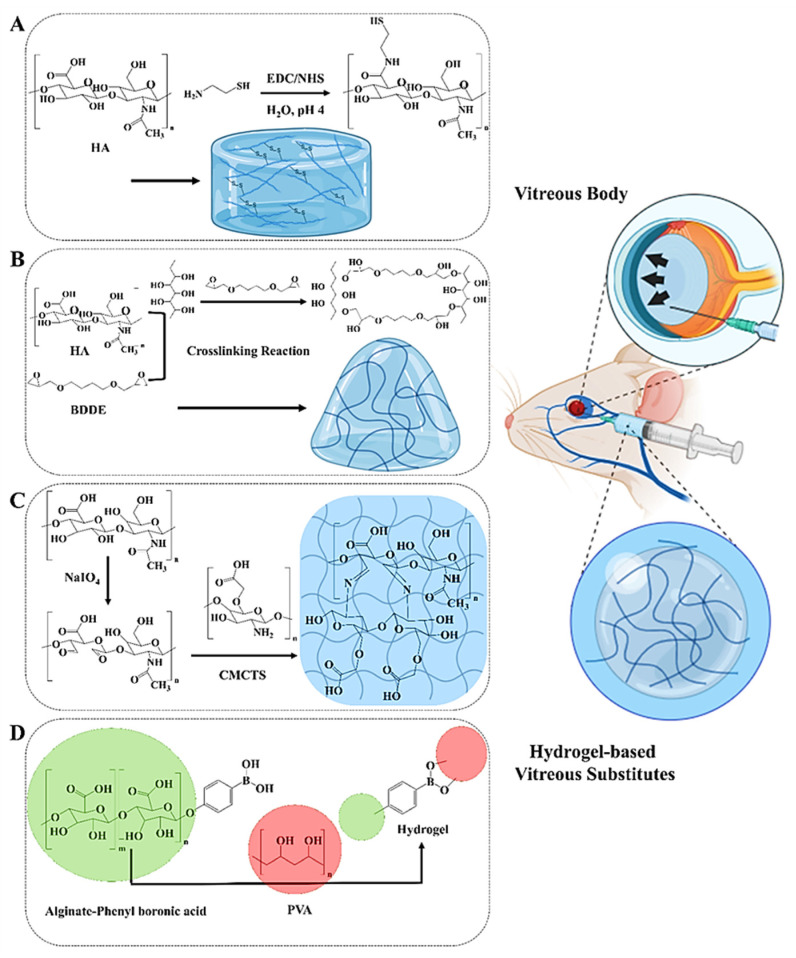
Illustration of natural-polymer-based hydrogel as a vitreous substitute: (**A**) SH-HA synthesis scheme, (**B**) schematic of HA-BDDE crosslinked hydrogel as a vitreous replacement, (**C**) injectable hydrogel based on water-soluble chitosan and hyaluronic acid for a vitreous substitute, and (**D**) in situ alginate-phenylboronic acid/polyvinyl alcohol composite hydrogel (TALPPH) as a potential vitreous substitute with a tamponading function.

After pars plana vitrectomy, Healon GV, UVHA, and SO-5000 were compared. Both forms of hyaluronic acid hydrogels were safe for humans and maintained therapeutic IOP levels (≥5 mmHg) for up to three months. UVHA affected IOP the longest. The typical six-month follow-up period saw no enucleations and improved or maintained eyesight. Optical coherence tomography demonstrated that UVHA-treated eyes exhibited fewer choroidal folds but intact retinal architecture. These findings suggest resorbable hyaluronic-acid-based hydrogels might treat phthisis bulbi instead of silicone oil. They are biocompatible, helpful, and do not need surgery [27]. Hyaluronic acid breaks down naturally, making it popular in biomedical and tissue engineering applications, according to research. Adding innocuous functional groups to hyaluronic acid slows its breakdown without affecting its properties. As shown in Figure 2A, Yu et al. [8] created biocompatible, transparent, swellable, and biodegradable hydrogels suitable for vitreous replacement. These hydrogels were synthesized by incorporating sulfhydryl groups derived from natural human tissues into hyaluronic acid (HA). By adjusting the degree of sulfhydryl substitution and the concentration of the polymer solution, the gelation time, swelling behavior, mechanical properties, and rheological characteristics of the hydrogels could be precisely tuned. The optimized SH-HA hydrogels are cell-friendly in cytotoxicity testing and demonstrate good histocompatibility and regulated in vivo biodegradability. A 90-day follow-up after vitrectomy showed normal intraocular pressure, B-ultrasonic diagnostics, fundus photography, OCT, and histology. Silicone oil is a common long-term vitreous replacement; however, SH-HA hydrogels may address its issues. Such outcomes may indicate fewer subsequent procedures and a better vitreous replacement [8].

Recent studies demonstrate that hyaluronic acid may mimic the physical properties of vitreous humor, making it a good substitute. As mentioned above, pure hyaluronic acid may cause inflammation following vitrectomy. Chen et al. [28] discovered that crosslinking hyaluronic acid with 1,4-butanediol diglycidyl ether (BDDE) to form HA hydrogels (HB) boosts anti-inflammatory properties (Figure 2B). To replace the vitreous body in eye procedures, the hydrogel (HBE) was treated with EGCG, an anti-inflammatory medication. HB was successfully created with a transparency, refractive index, and osmolality identical to native vitreous humor and could be readily injected. HBE significantly reduced inflammation by reducing inflammatory gene levels in retinal pigment epithelial cells compared to HB [28].

Incorporating nanoparticles into polymeric hydrogels has shown great potential for developing advanced drug delivery systems in the treatment of retinal diseases. Lopez et al. [29] utilized agar–hyaluronic acid hydrogels designed as vitreous substitutes to monitor the diffusion of nanoparticles (NPs) without fluorescent labeling, using caustic optical microscopy. Their findings revealed that NP diffusion within the hydrogel matrix is strongly influenced by particle size, highlighting the importance of size optimization in designing effective nanoparticle-based retinal therapies. Interestingly, the surface charge of the NPs did not significantly impact their distribution or diffusion within the synthetic hydrogel. This label-free imaging approach offers a cost-effective and reliable platform for evaluating novel therapeutic agents in vitro [29].

Schramm et al. [30] evaluated the potential of crosslinked carboxymethylated hyaluronic acid (CHA) hydrogels as vitreous substitutes. The hydrogels were crosslinked using ultraviolet (UV) light and N-vinyl-pyrrolidone and compared with those crosslinked via adipic acid dihydrazide (ADH). Both formulations were optically clear and transparent, with refractive indices comparable to that of the native human vitreous. Rheological assessments confirmed that the hydrogels exhibited viscoelastic properties suitable for intraocular application. While the ADH-crosslinked hydrogels demonstrated mild cytotoxicity toward retinal pigment epithelial (RPE) cells in vitro, the UV-crosslinked hydrogels showed no adverse effects. In vivo studies in a rabbit vitrectomy model demonstrated that the UV-crosslinked HA hydrogels were well tolerated over a six-week period, with no observed tissue damage [30].

In a related study, Barth et al. [31]. (2016) investigated Healaflow^®^, a crosslinked sodium hyaluronate hydrogel, as a synthetic vitreous substitute in a rabbit vitrectomy model. The hydrogel was applied during standard surgical procedures and remained optically clear postoperatively, although it experienced a notable reduction in volume over time. Despite surgical inconsistencies, electroretinography (ERG) confirmed that retinal function was preserved. Histological analysis revealed intact retinal architecture and activated Müller glial cells, with no significant DNA fragmentation. These findings suggest that Healaflow^®^ may serve as a safe and effective short-term intraocular tamponade [31].

Suri et al. [32] evaluated a natural polymer-based in situ hydrogel for vitreous replacement composed of gellan and hyaluronic acid at a ratio of 8:2 (*w*/*w*). Gellan gum, potentially substituting vitreous substance, solidified at ambient temperature and maintained its form at physiological temperature. Nevertheless, its mechanical and rheological properties limit its use. The crosslinking density was increased to improve gel properties. A hydrogel with robust crosslinks was synthesized using gellan gum, hyaluronic acid, and calcium chloride.

In another study, Laradji et al. [21] tested a thermo-sensitive hydrogel vitreous replacement that looks and works like the real vitreous body in terms of its physical, mechanical, and optical properties. The replacement comprises two parts: thiolated gellan, which is like collagen, and poly(methacrylamide-co-methacrylate-co-bis(methacryloyl)cystamine). At 45 °C, the hydrogel may be given as a thick solution. When it cools to room temperature, it quickly turns into a gel. Reversible disulfide crosslinking makes things stable and makes it easier to remove things in a controlled way. Two of the formulations acted like natural vitreous in terms of how they swelled, changed shape, and changed their refractive index, density, and optical transparency. After four months of looking at rabbit eyes after vitrectomy, there were no negative consequences, such as inflammation, cataracts, retinal detachment, or changes in intraocular pressure. Tests performed in vitro with retinal and fibroblast cell lines showed that the material is very biocompatible [21].

If its biophysical properties are optimized to align with those of the vitreous body, HA gel may serve as a viable short-term substitute. The short degradation period and ineffectiveness as intraocular tamponades limit their use. Polysaccharides, excluding HA, have been used as vitreous substitutes in limited research. Chitosan is being investigated as a vitreous substitute because of its physiological similarities. In rabbit eyes, intravitreal tamponade using chitosan did not impact intraocular tissues or pressure. Chitosan implants are superior to HA implants for vitreous cavity filling due to their analogous inflammatory responses and extended biodegradation period. Chitosan is a polysaccharide produced by removing acetyl groups from chitin. It is non-toxic and breaks down spontaneously, making it attractive in ocular biomaterials. However, native chitosan does not dissolve efficiently in the body, making it less effective in medicine [33].

As illustrated in Figure 2C, Wang et al. [34] developed an injectable hydrogel from dynamic Schiff base reactions that crosslink carboxymethyl chitosan (CMCTS) and oxidized hyaluronic acid (OHA). The CMCTS-OHA hydrogel has similar physical and chemical properties to natural vitreous. It can heal, remain stable, and transmit a high amount of light. The hydrogel’s water content (98.1%), refractive index (1.336), and density (1.005 g/mL) were similar to those of human vitreous. The optimized formulation, CMCTS-OHA3, offers balanced mechanical strength, compressive flexibility, shock absorption, and controlled swelling. Laboratory and animal tests demonstrated that the hydrogel was safe for cells and tissues and degraded slowly. A 90-day rabbit model demonstrated no adverse consequences from vitrectomy. These findings indicate that CMCTS-based hydrogels may be suitable for the next generation of biodegradable vitreous replacements that may replace synthetic and silicone-based agents in vitreoretinal surgery [34].

Chitosan is biocompatible, biodegradable, mucoadhesive, and electrostatically interacts with negatively charged ocular tissues, making it a preferred drug delivery method. Native chitosan does not gel and only breaks down in acidic situations [35]. Dextran, alginate, and chondroitin sulfate have been investigated as vitreous substitutes in both animal and human models. It has been discovered that these compounds induced little or no irritation. Nonetheless, their rapid breakdown, low viscosity, and inadequate tamponade efficacy make the vitreous substitutes ineffectual [9].

Collagen and its derivatives, including gelatin, have been evaluated for vitreous substitution. Since the 1960s, rabbits and people have received injections of polygeline, a degraded gelatin polypeptide, into their eyes. The chemical is non-toxic, elicits no tissue reactions, and often facilitates rapid retinal reattachment [36,37]. Polygeline served as a temporary vitreous substitute, akin to HA materials, due to its brief retention period in the vitreous cavity. Post-vitrectomy, rabbit vitreous was infused with collagen and hyaluronic acid to replicate normal vitreous conditions. The incorporation of HA into collagen prolonged the half-life of composite materials, and after three months, the implants did not adversely affect the ocular tissue [38]. New Zealand rabbits were administered methylated collagen (type I/III) to evaluate its ocular safety. Rabbit ocular tissues demonstrated compatibility with methylated collagen, indicating its safe implantation in the vitreous cavity without causing retinal injury [39].

Natural polymers used as vitreous substitutes have several disadvantages. Most natural polymers are unsuitable for inferior retinal tamponade because of their lower density compared to water. The primary issue with biopolymer-based methods is rapid deterioration. Even with crosslinking, these biomaterials gradually deteriorated, and their physico-mechanical characteristics diminished. HA implants have varying biodegradation rates influenced by their source, molecular weight, and chemical makeup, with the majority persisting for two to five months [40]. In contrast to biocompatible HA, collagen elicits a pronounced inflammatory response and moderate-to-severe ocular pain. Collagen gels often rupture post-injection, resulting in a loss of their structural integrity or functionality [41].

Alginate is a promising biomaterial for repairing both the vitreous body and cornea due to its favorable properties for vision restoration. Naturally transparent, biocompatible, and non-immunogenic, alginate offers a reduced risk of rejection and promotes graft integration. Its chemical structure can be easily modified or functionalized to enhance mechanical strength, biodegradability, or cellular interaction, making it adaptable for various ophthalmic applications. Of particular interest is its potential for corneal regeneration and vitreous substitution [5,42]. Choi et al. [43] developed a novel injectable hydrogel, TALPPH, composed of polyvinyl alcohol and alginate-phenylboronic acid, as illustrated in Figure 2D. This in situ-forming, self-healing, and transparent hydrogel closely mimics the viscoelastic properties of native vitreous. Its rapid gelation and ability to distribute microbubbles allow for minimally invasive injections through fine-gauge needles. In a rabbit model of retinal detachment, TALPPH provided effective retinal stabilization, prevented re-detachment, and preserved visual function over an extended period. Importantly, in vitro testing demonstrated no cytotoxicity to retinal cells, highlighting their biocompatibility. These features suggest TALPPH could serve as a next-generation alternative to conventional tamponades like silicone oil [43].

In a separate study, Wang et al. [44] engineered an alginate-based hydrogel inspired by biological systems to mimic the mechanical and optical properties of human vitreous. This translucent and elastic hydrogel demonstrated long-term safety and efficacy in rabbit eyes, maintaining intraocular pressure, minimizing inflammation, and preserving retinal function. Despite some brittleness, the hydrogel remained securely positioned in the vitreous cavity after injection and gelation. Collectively, these findings underscore alginate’s potential as a long-term vitreous substitute. With its tunable mechanical properties, excellent biocompatibility, and optical clarity, alginate-based hydrogels represent a promising platform for future ophthalmic therapies and ocular tissue engineering applications [44].

### 2.2. Synthetic Polymers

Synthetic polymers are garnering significant interest in ocular restoration due to their ability to modify mechanical characteristics, undergo controlled degradation, be reactivated, and be readily functionalized. Synthetic polymers have superior longevity, enhanced stability, and greater purity compared to natural polymers. These are essential for ocular applications, including corneal repair, vitreous replacements, intraocular lenses, and drug delivery systems. Polymeric hydrogels are ideal for vitreous replacement. Crosslinking hydrophilic polymers that absorb water results in their expansion and the creation of a gel network [45].

Poly(2-hydroxyethyl acrylate) (PHEA), polyacrylamide (PAA), and poly(1-vinyl-2-pyrrolidone) (PVP) showed failure attributed to inflammation and toxicity, despite their advantageous transparency and rheological characteristics [46]. HPMC and PGMA were evaluated; however, their short degradation durations limited their use in clinical studies [47,48]. Notable polymers include PVA, PVA-MA, PHA, PHEMA, PAA copolymers, and poly (vinyl alcohol) methacrylate. These polymers have enhanced biocompatibility, transparency, and rheological properties; nevertheless, more clinical studies are required to evaluate their long-term durability and compatibility [49,50,51,52].

Polymeric hydrogels may deliver medications and nutrients to promote healing and provide tamponade. The gel-like nature of many polymers compromises their structural integrity when injected with a small-gauge needle [53]. The inability to inject thirty-eight PHEMA into a smallbore aperture during vitreous–retinal surgery necessitates a bigger surgical incision, increasing procedural complexity and the risk of ocular damage [54].

Several crosslinkers and initiators, whether chemical or physical (e.g., light or oxygen), may be integrated into soluble monomers to produce gels in situ. Disulfide crosslinkers are acknowledged for their capacity to link acrylamide monomers during atmospheric oxidation. This research synthesized disulfide-bonded acrylamide hydrogels using free-radical polymerization in aqueous ethanol [55]. The hydrogels were solubilized with dithiothreitol to yield water-soluble acrylamide copolymers with pendant thiols. The hydrogels were reconstituted by exposing thiol-containing copolymer aqueous solutions to 3,3′-dithiodipropionic acid or ambient oxygen. Swift endo-capsular gelation resulted in a transparent lens capsular bag gel. Despite the advantageous rheological characteristics and biocompatibility of in situ gels, acrylamide monomers have been linked to increased cancer risk [56].

Unreacted monomers may cause inflammation. The gelation process was projected to take one hour. A photo initiator can polymerize PVA-MA into transparent hydrogels. With an increase in methacrylate content, this methacryloyl derivative rendered PVA more hydrophobic, yet the polymer backbone retained sufficient hydrophilicity to facilitate hydrogel formation during radiation exposure. The concentration of photo initiators and the duration of exposure may influence crosslinking. Inadequate crosslinking caused hydrogel degradation. Consequently, PVA-MA hydrogels characterized by elevated crosslinking and polymer content were shown to be the optimal options. Nonetheless, these gels exhibited far greater stiffness than typical vitreous. Moreover, there is a lack of data to substantiate the efficacy of UVA wavelength light in surgical procedures, and a comprehensive assessment of PVA-MA’s in vitro and in vivo characteristics is essential to explore vitreous biomimicry and biocompatibility [51].

Feng et al. [25] observed that the foldable capsular vitreous body (FCVB) improves the stability and preservation of PVA hydrogel inside the vitreous cavity. The researchers advocated for the advancement of the FCVB alongside other hydrogels, like PVP or PAA tamponade. The mechanical integrity and degradation of structures may be influenced by crosslinking density. Integrating pharmaceuticals into hydrogels may provide physicians with a sustained-release delivery system. Poly (ethylene oxide) aqueous solutions were evaluated in vivo as vitreous replacements in an animal model [22]. Aqueous solutions containing 5 wt% of 400 kDa poly (ethylene oxide) demonstrated viscoelastic properties similar to those of natural vitreous. Retinal cells integrated these medicines. Histology and electrophysiological parameters were stable, save for a 6-week increase in glial fibrillary acidic protein (GFAP) expression. A crosslinked poly (ethylene oxide) hydrogel may function as a superior artificial vitreous replacement, since the solutions did not remain in the posterior region postoperatively. A novel injectable PEG-based intraocular vitreous replacement was disclosed in different publications [57]. This gel employs hydrophobic thermo-sensitive poly (ethylene glycol). Increased polymer concentrations may provide flower-like micellar fluids with increased viscosity. This secure polymer swiftly gels by the creation of densely linked hydrophobic domains inside the vitreous cavity at physiological temperature. The gel is clear. It protects the retina and transmits light to the posterior sensory regions. Post-surgery, normal intraocular pressure was observed.

Table 2 presents a comparative overview of natural and synthetic polymers commonly employed in the development of hydrogel-based vitreous substitutes. It highlights each polymer’s physicochemical properties, biomedical advantages, limitations, and specific relevance to vitreous replacement. This comparison aids in understanding material selection strategies for optimizing biocompatibility, mechanical stability, and long-term performance in ocular applications.

## 3. Crosslinking Strategies in Hydrogel Design

**Figure 3:**
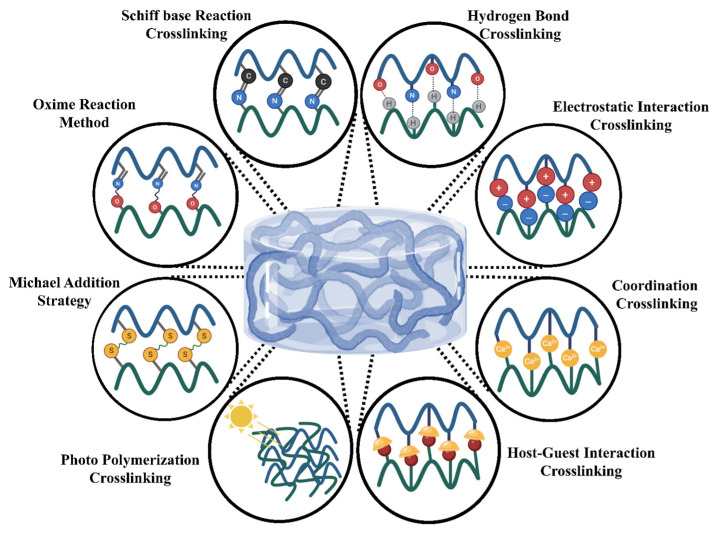
Illustration of various crosslinking strategies in hydrogel design via physicochemical crosslinking methods.

As illustrated in Figure 3, there are many physicochemical ways to crosslink polymers. The material’s qualities determine the procedure. There are many basic techniques to combine monomers or polymers with crosslinking agents to generate hydrogel polymeric networks [68]. Physical and chemical crosslinking procedures are the primary kinds. The crosslinking strategy used affects the hydrogel’s mechanical characteristics, swelling behavior, and biocompatibility. For biological or industrial purposes, choosing the proper technique is crucial [69,70].

### 3.1. Physical Crosslinking Methods

Physical crosslinking mechanisms employ dynamic, non-covalent interactions, enabling reversible network formation in polymeric systems. These include (1) hydrogen bonding between polar functional groups, (2) ionic complexation through electrostatic interactions, (3) physical entanglement of polymer chains, (4) crystalline domain formation, and (5) aromatic π-π stacking interactions [69].

**Hydrogen bonding**: Nature contains many hydrogen bonds. Functional and safe medical hydrogels are being developed by scientists. Functional groups that exchange hydrogen affect hydrogen bonding between gel-forming polymers and monomers. Hydrogen bonds may crosslink hydrogels depending on the solvent, component molar ratio, reaction temperature, and polymer concentration [71].

Uesugi et al. [72] (2017) developed Panacea Gel SPG-178, a self-assembling peptide hydrogel designed as a vitreous substitute. The hydrogel forms a nanofibrous network through hydrogen bonding and β-sheet interactions. At a 0.1% peptide concentration, the gel exhibited 96.7% visible light transmission and a refractive index of 1.3339, both closely matching those of natural vitreous. Upon mechanical disruption, the gel became shear-thinning and injectable through a 27-gauge needle. Rheological analysis confirmed gel-like viscoelasticity, with a storage modulus (G′) of 18.12 Pa at 1 rad/s. The β-sheet structure remained stable due to reversible, non-covalent hydrogen bonds. In a rabbit vitrectomy model, the hydrogel remained transparent and structurally stable for up to three months, without inducing intraocular inflammation, retinal toxicity, or intraocular pressure abnormalities, as confirmed by electroretinography and histological evaluation. Notably, this hydrogel does not require chemical crosslinking, making it a biocompatible and minimally invasive platform for long-term vitreous replacement [72].

In another study, Wang et al. [73] (2018) introduced a supramolecular binary copolymer hydrogel (PNAGA-PCBAA), formed by N-acryloyl glycinamide (NAGA) and carboxybetaine acrylamide (CBAA) monomers, which interact via hydrogen bonding. The hydrogel consists of 98.4% water and 1.6% polymer, with a refractive index of 1.336, a transparency of ~97%, and a modulus ranging from 1 to 10 Pa, closely mimicking the mechanical and optical properties of the native vitreous. It exhibits shear-thinning behavior, allowing injection through a 22-gauge needle, followed by rapid self-healing via hydrogen bond reformation. In vivo studies in rabbits demonstrated long-term biocompatibility for up to 16 weeks, with no signs of inflammation, retinal damage, or fibrosis. The zwitterionic CBAA component contributed to antifouling properties and minimized immune responses. Compared to traditional vitreous substitutes that suffer from instability, biofouling, and risk of fragmentation, this hydrogen-bonded system offers a stable, injectable, and self-healing alternative for vitreous replacement [73].

**Ionic/electrostatic forces**: Hydrogels formed through ionic and electrostatic interactions leverage complementary charged functional groups to create physically crosslinked networks [74]. Kim et al. [75] (2012) developed a thermo-responsive hydrogel composed of chitosan and β-glycerophosphate (GP), utilizing electrostatic interactions between the positively charged amine groups of chitosan and the negatively charged phosphate groups of GP. This hydrogel remains a low-viscosity solution at room temperature, enabling easy injection, and undergoes sol–gel transition at physiological temperature (37 °C), forming a stable network in situ. Increasing GP content (10–30 wt%) enhanced ionic interactions, evidenced by a decrease in zeta potential from +51 mV (chitosan alone) to +4 mV at 30% GP, with a corresponding increase in particle size from 400 nm to 1070 nm, indicating stronger intermolecular attraction and network density. Higher GP concentrations (20–30 wt%) resulted in slower gelation but improved long-term stability. The optimized formulation, CGP-30, enabled sustained release of bovine serum albumin (BSA) for up to two weeks, while CGP-20 released 10% of the payload within 24 h, and CGP-35 released 35% in the same period. This electrostatically crosslinked system demonstrates tunable gelation and release kinetics, making it a promising vehicle for intraocular protein delivery and vitreous substitution, especially for therapies requiring prolonged retention and controlled release [75].

**Hydrophobic interactions**: Heating induces hydrophobic interactions that facilitate the formation of hydrogels through the sol–gel transition. Micelles transform into hydrogels upon phase change due to heat, aggregating as a result of their hydrophobic nature. Liu et al. [76] (2021) developed dual physically crosslinked (DPC) hydrogels capable of self-healing through the formation of hydrophobic interactions. The mechanical properties of these hydrogels were also enhanced. A step in the procedure involves mixing N,N-dimethylacrylamide (DMAc) with DMAPMA-C18, a hydrophobic monomer characterized by an alkyl chain of C18. The core network consists of C18 chains that exhibit hydrophobic properties. CNC and CNC-C8 serve as effective nanofillers and secondary crosslinkers. The C18 and C8 chains exhibit hydrophobic interactions alongside hydrogen bonding and electrostatic forces. The CNC-C8 DPC hydrogel exhibits superior stretchability (4268% ± 1446%) and load-bearing capacity (331 ± 32 kPa) compared to non-hydrophobized CNC hydrogels. Tetrahydrofuran (THF) can be employed rapidly for the repair of hydrogels without compromising their mechanical integrity. This article discusses the potential of manufacturing hydrogels to produce materials that exhibit strength, flexibility, and self-healing properties through hydrophobic interactions. This presents new opportunities for enhanced applications in biology and technology [76].

**Crystallization**: Crystallization is a common way to crosslink hydrogels. Freezing and thawing polyvinyl alcohol are two steps in making hydrogels. As hydrogel freezes and turns into ice crystals, the phases are clearly defined. The higher the concentration of PVA chains, the more hydrogen bonds are formed and the more crystalline domains generated, which are physical crosslinking sites in the hydrogel network. Crystalline parts make the gel’s structure stronger when it is thawing, which makes it stronger and less likely to swell. Annealing after many freeze–thaw cycles improves the crystallinity by 20% to more than 60%. Both the gel fraction and the strength become stronger. Chemicals are not needed for crosslinking that is based on crystallization. This results in stable hydrogels that are safe to use on people. Changing the concentration, freezing time, cycles, and annealing temperature changes the properties of polymers. These hydrogels have reversible physical crosslinks that are mostly made up of hydrogen-bonded crystalline domains. This makes them strong, porous, and useful for drug delivery and tissue engineering [77].

The structure of hydrogel networks greatly influences their mechanical and functional properties. Raia et al. [78] (2020) explored silk–hyaluronic acid (HA) composite hydrogels as potential vitreous substitutes. Their findings showed that varying the silk/HA ratio and crosslinking density affected hydrogel stiffness and stability. The hydrogels achieved a refractive index of 1.336 comparable to natural vitreous and exhibited an optical transparency of between 75 and 91%. Silk’s ability to form β-sheet crystals contributed to physical crosslinking, enhancing structural integrity over time. Increased silk content promoted β-sheet formation, resulting in improved mechanical strength and shape recovery. Dynamic light scattering confirmed a dense, interconnected network suitable for molecular diffusion. Intraocular pressure tests in porcine eyes demonstrated that the hydrogels maintained pressure similarly to silicone oil. These results suggest that silk–HA hydrogels offer a promising, biocompatible, and mechanically resilient alternative to current vitreous substitutes [78].

**Host–guest interactions:** Host–guest hydrogels are widely studied due to their tunability, ease of formation, and reversible non-covalent interactions. These systems rely on the inclusion of guest molecules within the cavities of host molecules, creating dynamic, physically crosslinked networks. This reversible interaction enhances network stability by minimizing unfavorable interactions with the external polar environment. Typically, host or guest moieties are incorporated into the polymer backbone via terminal or side-chain modifications to promote intermolecular crosslinking. However, placing both on the same chain can lead to intramolecular loops rather than network formation [79,80].

Fang et al. [81] (2025) developed a pseudo-poly-rotaxane hydrogel (DEX-NPH) by combining γ-cyclodextrin (γ-CD) with Tween 80-based nano emulsions. The host–guest interactions reduced corneal irritation and enabled sustained drug delivery. The hydrogel displayed thixotropic and shear-thinning behavior, making it suitable for ocular application. Modulating the crosslinking between γ-CD and PEG chains influenced gel formation and drug release. Pharmacokinetic analysis revealed that DEX-NPH significantly prolonged the corneal half-life and bioavailability of dexamethasone. In a rat model of alkali-burn-induced ocular inflammation, the hydrogel outperformed free drug and nano-emulsion alone in therapeutic efficacy. Moreover, ocular safety assessments showed no signs of irritation. These findings highlight host–guest-based pseudo-poly-rotaxane hydrogels as promising platforms for treating inflammatory eye diseases through prolonged and targeted drug delivery [81].

### 3.2. Chemical Crosslinking Methods

Some well-known chemical crosslinking processes may create persistent covalent networks. These include heat- or light-induced free-radical polymerization, enzyme-aided conjugation processes, high-energy-radiation-induced bond formation, and multifunctional crosslinking agents that connect reactive groups on polymer backbones. These covalent bonding technologies provide stronger, more robust networks than physical crosslinking systems. Chemical crosslinking creates hydrogels in many ways, including Michael additions, oxime reaction, Schiff base synthesis, and oxidation and salting-out methods [69].

**Polymerization technique:** Hydrogels are formed by linking monomers or macromers to create networks that incorporate water. Common processes encompass free radicals, photopolymerization, and biological life. These strategies alter their strength, structural composition, and rate of degradation. Regulating gelation in photopolymerization is crucial for applications in tissue engineering and ocular health. Functional crosslinkers or stimuli-responsive units may enhance medicinal applications [82,83]. Li et al. [84] (2025) formulated a photocurable dual-network hydrogel utilizing OCS and GelMA. Hydrogel may serve as an alternative to stitches for the healing of large corneal ulcers. The hydrogel is polymerized through the interpenetrating network method. It exhibits increased strength, adhesion, and resistance. The process involves the combination of amino groups from GelMA with OCS aldehyde, followed by the application of visible light to facilitate the crosslinking of GelMA. This hydrogel is transparent, adhesive, and non-expanding, making it suitable for application on the cornea. Studies on rabbits indicate that this therapy reduces swelling, regenerates stroma, and addresses large corneal ulcers. In vitro, studies demonstrated that human corneal epithelial cells exhibited superior growth, motility, and adhesion compared to other cell types. Hydrogels utilizing both polymerization-based crosslinking and natural biopolymer chemistry may facilitate corneal tissue engineering and healing without the need for sutures. These hydrogels exhibit durability and biocompatibility [84].

**Enzymatic crosslinking:** Enzymatic crosslinking offers a biocompatible and cell-friendly approach for hydrogel fabrication, operating efficiently at low temperatures and under physiological conditions. This method relies on enzymes to catalyze covalent bonding between functional groups on polymer chains, enabling in situ gelation. For example, tyrosinase oxidizes tyrosine or catechol groups to form crosslinks, while horseradish peroxidase (HRP) facilitates coupling of phenol or tyramine groups in the presence of hydrogen peroxide. Transglutaminase catalyzes amide bond formation between glutamine and lysine residues in protein-based polymers like gelatin [85]. These systems allow precise control over crosslinking density and gelation time by adjusting enzyme or substrate concentrations. Unlike chemical crosslinking, enzymatic methods are non-toxic and well suited for ocular applications such as injectable adhesives, corneal scaffolds, and drug delivery systems [86,87].

For instance, Hou et al. [88] (2018) developed an injectable microporous hydrogel by enzymatically crosslinking gelatin microgels (250 µm diameter) using microbial transglutaminase (mTG). The resulting bulk hydrogel contained interconnected pores that promoted rapid migration and proliferation of human dermal fibroblasts over two weeks, while preserving viscoelastic properties similar to conventional gelatin hydrogels. When injected into ex vivo porcine corneal defects, the hydrogel adhered well, prevented cell infiltration from surrounding tissues, and provided sustained release of platelet-derived growth factor to support tissue regeneration. This enzyme-crosslinked microgel system shows promise for tissue engineering and wound healing applications, where controlled cell infiltration and growth factor delivery are essential [88].

**Michael addition strategy:** Michael addition is a widely used crosslinking strategy for hydrogel formation that is particularly valuable in ocular drug delivery and tissue engineering. This nucleophilic addition reaction occurs when donor groups such as thiols (–SH) or amines (–NH_2_) react with electron-deficient acceptors like acrylates, maleimides, or vinyl sulfones. A key advantage is its compatibility with aqueous, physiological conditions without the need for harmful catalysts or initiators, which is ideal for encapsulating sensitive biological agents such as cells, proteins, and drugs [89].

Common linkages like thiol–acrylate and thiol–maleimide yield robust hydrogel networks with tunable mechanical properties and degradation profiles. The mild reaction conditions and biocompatibility make Michael addition particularly suitable for in situ-forming injectable hydrogels and ocular bioadhesives, offering precise control over crosslinking [90].

For example, Chang et al. [91] (2015) developed a zwitterionic hydrogel via Michael addition for use as a long-term vitreous substitute. The hydrogel was synthesized from poly (MPDSA-co-AC) copolymers, where acryloyl cystamine introduced thiol groups and sulfobetaine methacrylamide imparted antifouling and biocompatibility. α-PEG-maleimide served as the crosslinker in a thiol–ene reaction, enabling rapid in situ gelation. The resulting hydrogels were optically clear, tunable in gelation and mechanical strength, and exhibited minimal swelling—key characteristics for ocular applications. In a rabbit model, hydrogels with a 2:1 thiol–ene ratio formed stable, transparent implants that remained biocompatible for at least two months. This work demonstrates the potential of Michael addition to generate minimally invasive, injectable, and durable hydrogels for vitreous replacement and soft-tissue repair [91].

**Oxime reaction strategy:** Oxime chemistry offers a biocompatible and precise approach for hydrogel crosslinking that is particularly suited for sensitive biomedical applications. This reaction involves the formation of oxime bonds (–C=N–OH) through the condensation of aminooxy (–ONH_2_) groups with aldehyde or ketone groups. Unlike traditional crosslinking methods, oxime formation occurs efficiently in aqueous environments under physiological temperature and pH, without the need for toxic catalysts, making it ideal for injectable systems, ocular adhesives, and cell-encapsulating hydrogels. Compared to imine (Schiff base) linkages, oxime bonds are more hydrolytically stable, enabling rapid and durable gelation under mild conditions. As such, oxime-crosslinked hydrogels represent a promising platform for long-term ocular surface applications and controlled drug delivery systems [92,93]. Baker et al. [94] (2021) demonstrated this approach by developing a hyaluronan-based hydrogel crosslinked via oxime chemistry to mimic the native vitreous body. In this system, hyaluronan was chemically modified with aldehyde and ketone groups, which were subsequently crosslinked using poly (ethylene glycol)-tetraoxyamine. This method enabled tunable gelation kinetics and produced a hydrogel with optical and physical properties (e.g., density and refractive index) closely resembling those of natural vitreous. The hydrogel exhibited minimal swelling, could be delivered through fine-gauge needles, and showed excellent safety for retinal photoreceptors in vitro. In vivo studies in rabbits confirmed that the oxime-crosslinked hydrogel maintained intraocular pressure, preserved retinal structure and function for up to 90 days, and underwent gradual biodegradation beginning around day 28. These findings underscore the potential of oxime chemistry for creating injectable, stable, and biodegradable vitreous substitutes with excellent biocompatibility, marking a significant advancement toward clinically viable retinal repair materials [94].

**Schiff base formation**: Schiff base chemistry is widely used for forming dynamic covalent bonds in hydrogel systems due to its simplicity, reversibility, and inherent biocompatibility. It involves the formation of imine bonds (–C=N–) through the reaction between primary amines and aldehyde or ketone groups. These reactions occur efficiently in aqueous environments at physiological temperatures, without the need for toxic initiators or catalysts, making them especially valuable for biomedical applications such as ocular adhesives, injectable gels, and tissue-engineered scaffolds. A notable feature of Schiff base linkages is their self-healing ability and responsiveness to pH. In acidic environments, imine bonds are prone to hydrolysis, leading to gradual degradation, particularly when compared to more stable linkages like oximes. Nevertheless, Schiff-base-crosslinked hydrogels offer rapid gelation, tunable mechanical properties, and compatibility with a wide range of polymers, supporting their application in corneal regeneration, wound repair, and targeted drug delivery [95,96].

Chand et al. [97] (2025) introduced a biomimetic strategy for engineering corneal stroma equivalents using a dual-crosslinked interpenetrating network (IPN) hydrogel that combines Schiff base chemistry with photopolymerization. The hydrogel is composed of oxidized carboxymethylcellulose (OxiCMC) and gelatin methacryloyl (GelMA). Dynamic imine bonds form between the aldehyde groups of OxiCMC and the amine groups of GelMA, while UV exposure induces secondary photopolymerization of GelMA, reinforcing the hydrogel structure. This dual-crosslinked system yields hydrogels with excellent optical clarity, high compressive strength (106.3 ± 7.7 kPa), and structural features closely resembling native corneal tissue. It is also compatible with digital light processing (DLP) 3D bioprinting, enabling precise fabrication. The printed constructs exhibit high porosity, interconnected architecture, and support over 93% viability of embedded human corneal keratocytes. These features make the system highly reproducible, biologically safe, and well-suited for corneal tissue engineering without relying on synthetic polymers or toxic crosslinkers.

**Oxidation and salting out:** Oxidation and salting-out represent an innovative approach to hydrogel fabrication, enabling the production of highly flexible, conductive polyvinyl alcohol hydrogels under mild conditions. In this method, ammonium persulfate (APS) serves a dual role as both an oxidizing agent and a salting-out agent. APS introduces carboxyl and aldehyde groups onto PVA chains by oxidizing hydroxyl groups, thereby enhancing hydrogen bonding and increasing the availability of reactive sites for intermolecular interactions. Simultaneously, ammonium and sulfate ions promote the salting-out effect, reducing PVA solubility and facilitating chain aggregation and crystallization. This dual chemical–physical crosslinking strategy allows rapid formation of a porous and highly interconnected hydrogel network, eliminating the need for traditional freeze–thaw cycling. Importantly, it enables the use of low-molecular-weight PVA (~30 kDa), offering greater flexibility and tunability in hydrogel design. Zhang et al. [98] (2025) demonstrated that this method yields OxPVA hydrogels (OxPVAHGs) with excellent elasticity, ionic conductivity, and mechanical strength, making them ideal for biomedical applications. These hydrogels can be customized in terms of conductivity and responsiveness, offering functional advantages for biomedical and ophthalmology.

## 4. Mechanical Properties of Hydrogel Vitreous Substitutes

The vitreous humor is a transparent, viscoelastic hydrogel occupying approximately 80% of an eye’s volume, playing a critical role in maintaining ocular shape, distributing mechanical stress exerted on the eye, and supporting the retina’s stability. Its gel-like consistency driven by a collagen–hyaluronic acid matrix enables it to act as both a shock absorber for the eye and structural filler in the eye [99,100]. In surgeries like vitrectomy, performed to treat retinal detachment, diabetic retinopathy, or ocular trauma, the native vitreous is removed. If not replaced with a biomechanically appropriate material, complications like elevated intraocular pressure (IOP), retinal detachment, or visual distortion may follow [101]. An ideal vitreous substitute must therefore replicate the physical and mechanical characteristics of the native vitreous. Table 3 summarizes the key requirements:

Perfluorocarbon liquids (PFCLs) offer advantages during vitreoretinal surgery due to their high density (1.76–2.03 g/mL) and strong surface tension (40–45 mN/m), which help in flattening and controlled positioning of the retina. However, they are nor suitable for long-term use in the eye due to retinal toxicity and risks of emulsification [78].

**Figure 4:**
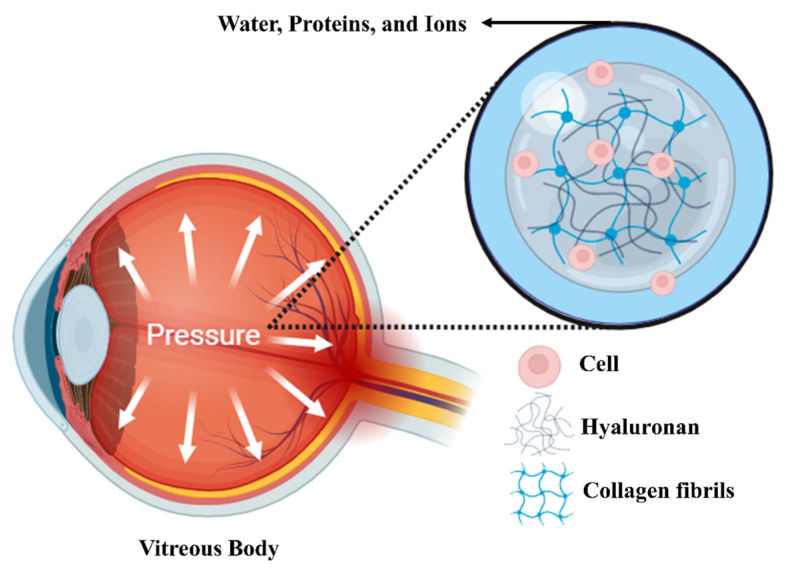
Diagram of the vitreous constituent parts in the human eye.

As shown in Figure 4, while the vitreous in the human eye is 99 percent water, collagen fibers and hyaluronic acid make up most of the remaining 1 percent. The mechanical characteristics of the vitreous are not greatly impacted by water, ions, or constituents like cells and proteins. Collagen fibers and hyaluronic acid are the main sources of the vitreous’s mechanical characteristics [94].

Hyaluronic-acid-based materials are widely recognized for their excellent biocompatibility and ability to replicate the viscoelastic behavior of the natural vitreous. However, if not stabilized, their rapid enzymatic degradation in physiological condition and the risk of intraocular pressure fluctuations limit their long-term performance [94]. These challenges highlight the need for advanced vitreous substitutes, particularly injectable hydrogels that combine long-term stability, controlled degradation, viscoelasticity, and suitable biocompatibility for long-term intraocular stability.

**Figure 5:**
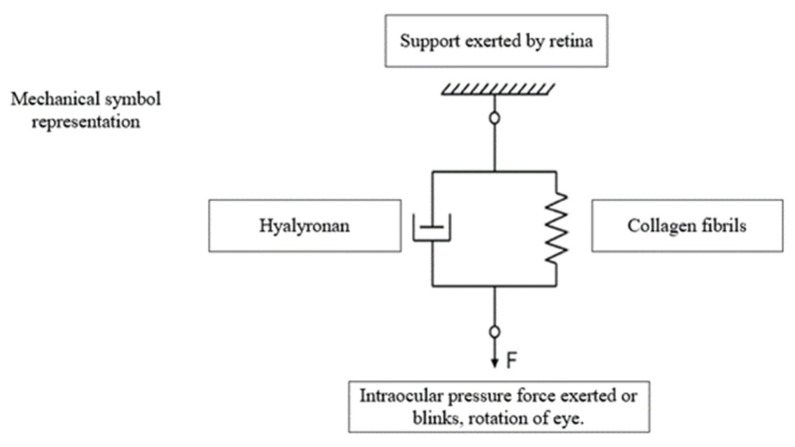
Schematic of the Kelvin–Voigt model illustrating hyaluronic acid’s viscoelastic behavior, with a spring for elasticity and a dashpot for viscosity. Internal pressure (IOP) applies to the force. Adapted from Figure 1 of [104].

As shown in Figure 5, the Kelvin–Voigt model uses a parallel spring and dashpot (damper) to represent viscoelastic materials. The spring represents hyaluronic acid’s elastic properties, which allow it to store mechanical energy and spring back after stress is released. The dashpot represents hyaluronic acid’s viscous properties, which cause energy loss over time. Viscoelastic solids are made of these components and resist sudden deformation and recover shape. This model is crucial for studying soft biological tissues or hydrogels like hyaluronic acid ones, where elasticity and viscosity affect mechanical behavior. Note that force here is actually exerted by internal pressure (IOP) [104].

### 4.1. Mechanical Characterization of Hydrogel-Based Vitreous Substitutes

A detailed mechanical characterization is essential to ensure that the vitreous substitute will mimic the functional behavior of natural vitreous, particularly in terms of its response to deformation. Two key rheological parameters will define the viscoelastic behavior of hydrogels: the storage modulus (G′) and the loss modulus (G″). The storage modulus (G′) shows the elastic nature of a material, its ability to store mechanical energy and recover its original shape after deformation like a spring. In contrast, the loss modulus (G″) reflects the viscous behavior of the material, which indicates the rate at which energy is dissipated as heat during deformation, like a damper [78]. The natural vitreous body exhibits both solid-like and fluid-like characteristics. Therefore, an ideal vitreous substitute should have a balance between elasticity and viscosity. Typically, the native vitreous shows a low G′ value ranging from 5 to 10 Pa. Replicating these soft viscoelastic properties is important to prevent complications such as elevated intraocular pressure (IOP) or abnormal mechanical stress on the retina [105]. The ratio between G′ and G″ is important before and after injection of hydrogel materials. Prior to implantation, a predominant viscous behavior (G″ > G′) improves smooth injection through fine-gauge needles (22–33 G). After injection, in situ gelation at physiological temperature (~37 °C) should transit the hydrogel to an elastic-dominant state (G′ > G″). This condition will help maintain its shape and provide mechanical support within the vitreous cavity. Thermo-responsive hydrogels like PYK-1105 and PEG-based hydrogels are designed to exhibit this reversible transition from viscosity-dominant to elasticity-dominant properties. These materials demonstrate shear-thinning behavior during injections, minimizing tissue trauma, and rapidly recover to a gel state at body temperature, offering stable tamponade properties [101,106].

Achieving an optimal mechanical property is critical not only for surgical condition but also for long-term performance in the eye. If the hydrogel is too stiff (G′ too high), it can make physiological eye movements harder and increase the IOP of the eye. On the other hand, if it is too soft (low G′ and G″), it may fail to maintain its form or may settle under gravity. Therefore, careful tuning of viscoelastic properties remains a fundamental aspect of developing safe and effective hydrogel-based vitreous substitutes [78]. The method of delivery for vitreous substitutes during vitreoretinal surgery is as critical as their long-term in vivo stability. A key factor influencing its injectability is the hydrogel’s response to shear stress. When injected through narrow-gauge needles (22–33 G), hydrogels experiences high shear forces. Depending on molecular structure and crosslinking density, they may exhibit shear-thinning or shear-thickening properties [106]. Shear-thinning hydrogels’ viscosity decreases under stress, leading to smoother flow through fine needles. This behavior is particularly advantageous in surgery conditions, as it reduces injection force and minimizes the risk of trauma to delicate intraocular structures. In contrast, shear-thickening materials increase in viscosity under shear stress, making injection harder and potentially requiring higher pressures that could disrupt gel integrity or damage retinal tissue [100]. The loss tangent (tan δ), calculated as G″/G′, provides information about a material’s viscoelastic behavior under injection shear stress. When tan δ > 1, this indicates viscous (liquid-like) dominance, while tan δ < 1 reflects elastic (solid-like) behavior. For optimal injectability and in situ performance, hydrogels are preferred to exhibit tan δ > 1 prior to injection, ensuring fluid-like behavior, and tan δ < 1 post-injection, signifying gelation and structural stability. This viscoelastic transition from a liquid state at room temperature to solid form at body temperature is a main characteristic property of in situ-forming thermo-responsive hydrogels. For instance, Yu et al. [107] demonstrated that a 3% PVA/chitosan hydrogel achieved good injectability, optical transparency, and long-term mechanical properties. After 24 weeks of implantation, the storage and loss module remained closely matched, indicating minimal degradation and sustained structural integrity over time. These findings highlight the importance of engineering the hydrogels with tailored rheological properties that support both smooth injection and durable tamponade performance after injection [107].

### 4.2. Aging and Dehydration Effects on Mechanical Stability

**Figure 6:**
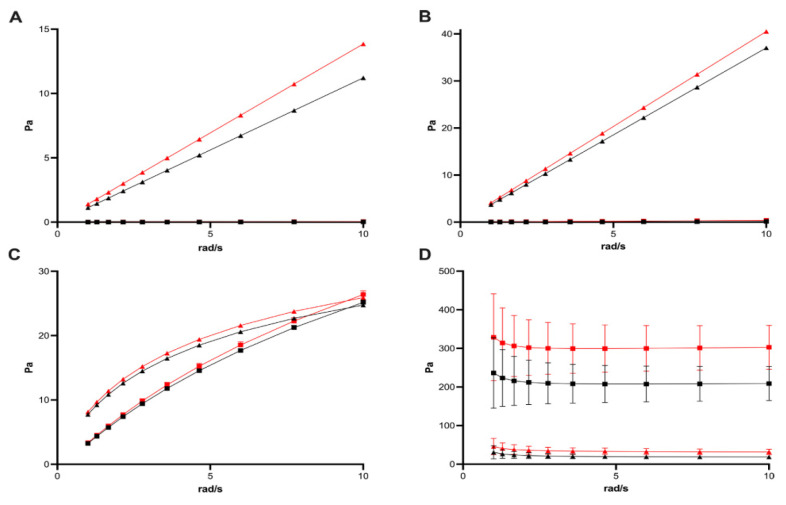
Effect of aging on different hydrogels for vitreous hydrogel substitutes. Dark lines are before, and red lines are after aging for (**A**) Densiron, (**B**) Siluron 5000, (**C**) Albomed HA, and (**D**) alginate 0.5%. Also, it is worth noting that these diagrams represent the elastic modulus numbers in Pa. As is clear after aging, the elastic modulus is increased, so this will result in greater stiffness, which could cause the pressure in the eye to rise [100].

Over time, environmental factors such as dehydration and thermal exposure can greatly change the mechanical properties of vitreous substitute hydrogels. One of the most noticeable changes observed in aging is increased stiffness of the hydrogel, typically resulting from water loss and structural rearrangement. As water evaporates from the hydrogel structure, whether during long-term storage or in vivo implantation, polymer chains may become more compact and crosslinking density may increase. This process elevates the storage modulus (G′), indicating heightened mechanical stiffness [100]. This stiffening trend has been observed in both commercial and experimental vitreous substitutes. Post-aging studies of Densiron, Siluron 5000, Albomed hyaluronic acid, and 0.5% alginate hydrogels demonstrated increased elastic modulus values compare to their fresh counterparts. As shown in Figure 6, all tested materials exhibit higher stiffness following aging experiments. While moderate stiffening of the hydrogel can help to maintain mechanical support on the retina, excessive rigidity can disrupt ocular biomechanics, elevate intraocular pressure (IOP), or exert mechanical stress on sensitive tissues. This phenomenon is often related to progressive dehydration, particularly in hydrogels lacking dynamic water-retentive networks or osmotic stabilization.

The native vitreous consists of about 98–99% water, and any substitute has to maintain similar hydrogel incompatibility and also maintain the physiological function throughout time. From a design perspective, a key criterion for long-term vitreous implants is ability to maintain a consistent viscoelastic property. Hydrogels that exhibit minimal changes in G′, G″, and tan δ after prolonged implantation are more likely to support tamponade function and avoid negative mechanical effects in the vitreous chamber. Studies on accelerated aging experiments that examine these factors are crucial for predicting the in vivo stability of the hydrogel material. Strategies to reduce age-related stiffening and enhance the functional lifespan of vitreous substitutes include the addition of hydrophilic stabilizers, the formation of a semi-interpenetrating network, and the use of self-healing mechanism [100,108].

## 5. Recent Advances in Smart Hydrogel Systems

In recent years, significant progress has been made in developing smart hydrogel systems that closely mimic the native vitreous and in addressing the limitations of traditional substitutes. These new hydrogels are engineered for injectability and in situ gel formation and are responsive to shear force and safe degradation over extended periods of time in the body. They also offer tunable mechanical properties specifically for ocular applications. Thermo-sensitive hydrogels are among the most studied materials. They remain liquid at room temperature and rapidly gel upon exposure to physiological conditions. For example, PHxEP hydrogels, as demonstrated by Xue et al. [101], can form stable gels at body temperature even at low concentrations (0.5% *w*/*v*), reducing the injection force while maintaining mechanical strength [101]. Similarly, PYK-1105, developed by Stryjewski et al. [106], combines modified polyvinyl alcohol and polyethylene glycol to create a stable gel minutes after injection. These hydrogels maintain intraocular pressure (IOP) within normal limits and fully degraded within 14 days, offering both short-term tamponade and biodegradability without dangerous residues [106]. In contrast, Baker et al.’s [94] hyaluronic acid (HA)–oxime hydrogels exhibit improved durability, with complete degradation after about 300 days, making them suitable for applications requiring extended support and gradual absorption [106]. Beyond biodegradability and thermal responsiveness, emerging systems emphasize self-healing and mechanical adaptability. Wang et al. [35] introduced a CMCTS-OHA hydrogel capable of withstanding significant stress and autonomously repairing itself. Importantly, the storage (G′) and loss (G″) moduli remained stable before and after self-healing, underscoring its mechanical resilience, which is essential for coping with intraocular movements and tamponade deformation [34].

Advanced macromolecular engineering of the hydrogels also enabled synthesis of injectable anti-fouling hydrogels like the PCB-OAA system developed by Binbin He et al. [109]. This hydrogel exhibits rapid in situ gelation, has strong mechanical properties, and demonstrates resistance to protein and cellular adhesion. These properties are important for reducing inflammation and fibrosis during extended implantation. Its viscoelastic recovery includes a quick elastic phase followed by a slower viscous phase, closely mimicking the native vitreous behavior [109]. Finally, Jin et al. [110] engineered novel biomolecular designs such as DNA supramolecular hydrogels, exhibiting a high storage modulus (G′ = 680 Pa), shear-thinning injectability, and self-healing capacity, while demonstrating biocompatibility in vivo for over two months. Despite their potential, challenges remain, like controlling stiffness and full understanding of degradation behavior before clinical application [110]. Overall, these advances mark a transition from passive structural fillers to functional, adaptive vitreous substitutes capable of replicating both the static and dynamic biomechanics of the eye. The integration of self-regulating, environment-responsive, and programmable features is rapidly transforming hydrogel design for ophthalmic applications.

## 6. Evolution of Intraocular Tamponade Agents in Clinical Practice

Vitrectomy, the surgical removal of the vitreous humor, has become a cornerstone in the management of various retinal pathologies, including vitreous hemorrhage, retinal detachment, and epiretinal membranes [111,112,113]. One of its primary objectives is to stabilize the retina and maintain intraocular pressure, particularly in cases of rhegmatogenous retinal detachment (RRD) and intraocular bleeding.

As shown in Table 2, the concept of intraocular tamponade has changed significantly since its introduction in 1911, when Ohm [114] first introduced intravitreal air injection following external drainage of subretinal fluid to treat RRD. Later, Arruga et al. [115] advanced this approach by combining intravitreal air with diathermy, showing more effective retinal reattachment through enhanced fluid drainage and stabilization [115]. In the early 1970s, Dr. Edward W.D. Norton [116] introduced sulfur hexafluoride (SF_6_) gas as an intraocular tamponade for managing giant retinal tears [116]. Compared to air, SF_6_ provided a longer-lasting tamponade effect, improving retinal reattachment outcomes. Perfluoropropane (C_3_F_8_) was later adopted due to its extended intraocular persistence, lasting approximately 55–60 days, in contrast to the 11–14 days typically observed with SF_6_ [117]. The choice between these gases often depends on the clinical context. For example, SF_6_ is preferred in primary cases, while C_3_F_8_ is typically used in more complex or recurrent cases due to its improved tamponade effect.

In a comparative study [118] involving 177 eyes with idiopathic macular holes, closure rates were similar for SF_6_ (86.4%) and C_3_F_8_ (86.5%), though C_3_F_8_ was associated with higher rates of intraocular pressure (IOP) elevation and cataract formation [118]. Moreover, in pneumatic vitreolysis (PV) for vitreomacular traction (VMT), SF_6_ was found to be a safer alternative to C_3_F_8_ due to its lower risk of IOP spikes and angle-closure glaucoma. A study [119] involving 30 eyes showed significant increases in IOP and persistent angle narrowing at 6 and 24 h postoperatively with C_3_F_8_, while SF_6_-related changes resolved by 24 h [119]. The use of liquid tamponades began with Paul Cibis and colleagues, who were the first to introduce silicone oil as a tamponade agent for retinal detachment repair [120]. Silicone oil, a hydrophobic compound composed of silicon–oxygen (Si–O) chains and hydrocarbon side groups, exhibits high viscosity and moderate interfacial tension, properties that enhance its intraocular stability and reduce the risk of emulsification [121]. Its long-term tamponade effect makes it particularly suitable for complicated cases such as proliferative vitreoretinopathy (PVR) or recurrent neovascularization [122]. The U.S. FDA approved silicone oil as a vitreoretinal tamponade in 1994 [123]. A 2021 meta-analysis highlighted silicone oil as an effective long-term tamponade for complicated retinal detachments [123]. A review study of 500 eye surgeries with silicone oil tamponade showed a 77% reattachment rate at six months after surgery [124]. Similarly, silicone oil removal (SOR) in 53 eyes showed improved best-corrected visual acuity (BCVA) and decreased IOP, although complications such as recurrent detachment (13.5%), hypotony (7.5%), and macular edema (49.1%) were reported within a one-year follow-up [125]. Silicone oil, while useful, is linked to various negative effects like cataract formation, glaucoma, keratopathy, choroidal thinning, and silicon-oil-related visual loss (SORVL) [126]. In a retrospective study of 65 eyes with giant-retinal-tear-associated RRD, no significant differences were observed in recurrence rates or surgical outcomes between gas and silicone oil tamponades [127]. However, another study noted irreversible thinning of the retina and subfoveal choroid following silicone oil tamponade in RRD patients, unlike in gas tamponade cases [128]. A meta-analysis further demonstrated that gas tamponade resulted in greater visual improvement, although both agents achieved comparable reattachment rates [129].

Perfluorocarbon liquids (PFCLs), introduced by Dr. Stanley Chang [130], are used as intraoperative instruments for stabilizing detached retinas, draining subretinal fluid, and handling traumatic injuries. PFCLs are favored for their high specific density and optical transparency, making them ideal for intraoperative retinal manipulation. In a study involving five patients with severe open globe injuries (OGIs), short-term PFCL tamponade (7–14 days) followed by replacement with silicone oil led to successful retinal and choroidal reattachment without major postoperative complications [131]. Similarly, in a study of 30 pediatric eyes, PFCLs used temporarily for approximately one week before replacement led to a high reattachment rate (91%) and functional visual improvement, with minimal complications [132]. However, PFCLs can be emulsified with silicone oil and must be thoroughly removed postoperatively to avoid adverse effects [133]. Comparative studies of single-stage versus two-stage pars plana vitrectomy (PPV) have shown better anatomical and visual outcomes with short-term PFCL tamponade followed by a secondary tamponade, rather than long-term silicone oil tamponade [134]. Despite their advantages, PFCLs carry a risk of retaining toxicity and require surgical expertise to ensure complete removal [135].

More recently, Vitargus^®^ (ABV-1701 Hydrogel) has emerged as a novel biodegradable hydrogel designed as a temporary vitreous substitute (clinical trial NCT04481386) (Table 4). Unlike traditional tamponades, Vitargus^®^ does not require surgical removal and provides uniform retinal tamponades without the need for postoperative face-down positioning. Phase I of the clinical trial, conducted between November 2016 and July 2018 at the Sydney Retina Clinic, involved 10 patients with RRD or vitreous hemorrhage. The trial demonstrated safety, good tolerability, no ocular toxicity, improved visual acuity, and stable retinal reattachment, laying the groundwork for subsequent clinical evaluations.

## 7. Challenges and Future Directions in Hydrogel-Based Vitreous Substitutes

Hydrogel-based vitreous substitutes represent a promising frontier in ophthalmology, offering the potential to closely mimic the native vitreous humor’s unique biomechanical and biological properties. However, despite significant advancements, several key challenges must be addressed to realize their full clinical potential. The vitreous humor is a highly specialized, transparent, viscoelastic gel composed predominantly of water, collagen fibers, and hyaluronic acid. Its mechanical role is vital; it maintains ocular shape, cushions the retina against mechanical stress, and facilitates nutrient transport. To replicate these functions, hydrogel substitutes must possess a finely tuned balance of viscosity and elasticity (viscoelasticity), appropriate density, and interfacial tension. Achieving this mechanical fidelity is complex because hydrogels must behave like a solid to support the retina yet flow like a liquid during injection. Parameters such as storage modulus (G′), loss modulus (G″), and loss tangent (tan δ) serve as crucial metrics for assessing whether the hydrogel mimics the dynamic mechanical behavior of the native vitreous across the injection and post-implantation phases [110].

A major practical consideration is the hydrogel’s injectability through fine-gauge needles (22–33 G) without damaging intraocular tissues. Smart hydrogel systems with shear-thinning properties allow viscosity to decrease under shear stress during injection but rapidly recover stiffness once in the vitreous cavity, providing both surgical ease and functional stability. Thermo-responsive hydrogels that remain liquid at room temperature and gel at physiological temperature have shown great promise in this respect [106,138].

One of the significant limitations of current hydrogels is their long-term mechanical and chemical stability. The native vitreous is highly hydrated (~98–99% water) and maintains its biomechanical properties over a lifetime. Hydrogels must similarly sustain their viscoelastic profile over extended periods to avoid complications such as retinal detachment or elevated intraocular pressure (IOP). However, issues like dehydration-induced stiffening, hydrogel degradation, and inflammatory responses pose challenges to sustain biocompatibility and mechanical function [100,108].

Hydrogels must not only replicate the mechanical environment but also be biologically inert or even supportive. Minimizing immunogenicity, inflammation, and fibrosis is essential to preserve retinal health and maintain optical clarity. Advances in anti-fouling hydrogel coatings, incorporation of bioactive molecules, and self-healing networks aim to enhance biological integration and reduce adverse responses [34,109]. Future hydrogel vitreous substitutes are expected to be multifunctional, incorporating stimuli-responsive [34,109] such as self-healing, on-demand degradation, and drug delivery capabilities. Hybrid hydrogels combining synthetic polymers with natural biopolymers, nanostructured reinforcements to improve mechanical robustness, and dynamic covalent chemistries that allow adaptive remodeling are key research avenues. These sophisticated materials could better emulate the vitreous dynamic responses to ocular movements and physiological changes [138].

Beyond scientific challenges, translation from bench to bedside requires rigorous biocompatibility testing, scalability of production under Good Manufacturing Practice (GMP) conditions, and robust clinical trials demonstrating safety and efficacy. Regulatory frameworks for these novel biomaterials must evolve to accommodate the complexity of smart hydrogels [106].

## 8. Summary and Outlook

Hydrogel-based vitreous substitutes are gaining attention as a next-generation solution for replacing the damaged or removed vitreous body in the eye.

With their high-water content, soft consistency, and excellent biocompatibility, these materials are well suited to replicate many of the natural vitreous functions. In this review, we have explored how different types of polymers both natural (like hyaluronic acid and collagen) and synthetic (such as polyethylene glycol and polyvinyl alcohol) can be used to engineer hydrogels with the right balance of transparency, flexibility, and biodegradability. We have also discussed how various crosslinking techniques influence the gel’s formation, strength, and long-term behavior.

While progress in this field is promising, some important challenges still need to be addressed. These include improving the long-term stability of hydrogels inside the eye, ensuring they integrate safely with surrounding tissues, and fine-tuning their mechanical properties to better support the retina. There is also growing interest in making these hydrogels more functional, for example, by loading them with drugs, designing them to respond to changes in the eye’s environment, or even helping promote tissue repair. Looking ahead, the future of hydrogel-based vitreous substitutes lies in interdisciplinary collaboration. Combining advances in materials science, chemistry, and ophthalmology will be key to translating these innovations into safe and effective treatments. With continued research and refinement, these biomaterials have the potential to significantly improve outcomes for patients undergoing vitrectomy or suffering from retinal diseases.

To further highlight the current barriers and future directions, Table 5 summarizes the most common challenges associated with hydrogel-based vitreous substitutes and outlines potential strategies to address them. This comparative overview provides a practical perspective on how ongoing innovations in polymer design, crosslinking chemistry, and biofunctionalization may overcome these limitations and enhance clinical translation.

## Figures and Tables

**Table 2:** Natural and synthetic polymers in hydrogel-based vitreous substitutes.

Type	Polymer	Hydrogel Properties	Advantages	Limitations	Ref.
Natural	Hyaluronic Acid	Viscoelastic, biodegradable, transparent	- High biocompatibility - ECM-mimetic - Anti-inflammatory	- Rapid degradation - Weak mechanical strength	[58]
	Collagen	Fibrillar structure, biocompatible	- Natural ECM protein - Supports cell attachment	- Low mechanical integrity - Risk of immunogenicity	[59]
	Gelatin	Thermo-sensitive, porous	- Biodegradable - Injectable - Functionalizable	- Rapid degradation - Limited mechanical durability	[60]
	Alginate	Ionically crosslinked, gel-like	- Mild gelation - Biocompatible - Injectable	- Poor mechanical strength - Not stable long-term in eye	[43]
	Chitosan	Cationic, bioadhesive	- Antibacterial - Biodegradable - Mucoadhesive	- Insoluble at neutral pH - May cause inflammation	[61]
Synthetic	PEG	Tunable, hydrophilic, crosslinkable	- Non-toxic - In situ gelation - Customizable degradation	- Lacks bioactivity - Requires crosslinkers	[62]
	PVA	Elastic, transparent, water-retentive	- High optical clarity - Stable structure - Biocompatible	- Non-degradable - Needs chemical crosslinking	[63]
	Poly(acrylamide) (PAAm)	Soft, transparent gel	- Mechanically stable - Tunable stiffness - Water-rich	- Not biodegradable - Monomer toxicity concerns	[50,64]
	Pluronic (Poloxamer)	Thermo-responsive triblock copolymer	- Injectable - Sol–gel transition near body temp	- Weak long-term stability - Low mechanical support	[65]
	Poly(N-isopropylacrylamide) (PNIPAM)	Thermo-responsive hydrogel	- Rapid gelation - Reversible phase transition	- Residual monomer toxicity	[66,67]

**Table 3:** Essential physical properties for an ideal vitreous substitute.

Property	Target Range	Description	Functional Importance	Ref.
Viscosity	300–2000 centipoise (cP)	Resistance to flow or thickness of the fluid	Allows for injection through small-bore needles while maintaining structure	[9]
Density	1.005–1.008 g/cm^3^	Mass per unit volume, matched to native vitreous	Prevents buoyancy-related displacement within the vitreous cavity	[102]
Viscoelasticity (Storage Modulus, G′)	~5–10 Pa	Gel-like elasticity and stress-response under deformation	Provides mechanical support and mimics the cushioning effect of natural vitreous	[103]
Buoyancy/Surface Tension	Sufficient interfacial tension	Floatation capability and sealing ability against retinal breaks	Facilitates tamponade of retinal breaks and stabilizes retina post-surgery	[17]
Injectability	Through 22–33-gauge needles	Compatibility with fine surgical needles	Enables minimally invasive delivery into the vitreous cavity	[34]
Shear-Thinning Behavior	Viscosity decreases under shear, recovers post-injection	Reduction in viscosity during injection with rapid gel recovery afterward	Facilitates easy injection while regaining mechanical strength in situ	[43]

**Table 4:** Overview of common vitreous substitutes used in clinical practice.

Product	Type	Clinical Use	Comments	Ref.
Air	Gas tamponade	Widely used for short-term tamponade	Oldest tamponade: short duration limits long-term use	[114,115]
Sulfur Hexafluoride (SF_6_)	Gas tamponade	Common primary tamponade for retinal detachment	Safer than C_3_F_8_ for IOP spikes; faster absorption	[116,119]
Perfluoropropane (C_3_F_8_)	Gas tamponade	Used for complex/recurrent retinal detachments	Longer tamponade effect but higher risk of IOP elevation and cataracts	[117,118,119]
Silicone Oil	Liquid tamponade	Long-term tamponade for complicated detachments	FDA-approved; associated with cataracts, glaucoma, keratopathy, and visual loss	[120,121,122,123,124,125,126]
Perfluorocarbon Liquids (PFCLs)	Liquid tamponade (intraoperative)	Short-term intraoperative tamponade	Used temporarily during surgery; must be removed to avoid toxicity	[130,131,132,133,134,135]
Vitargus^®^ (ABV-1701 Hydrogel)	Hydrogel-based substitute (investigational)	Under clinical trial for temporary vitreous substitution	Emerging hydrogel alternative with promising early clinical results	[14,136,137]

**Table 5:** Challenges of hydrogel-based vitreous substitutes and potential solutions.

Remaining Issues	Description	Potential Solution
Long-term stability	Hydrogels tend to degrade, shrink, or lose transparency over time inside the ocular environment	Designing interpenetrating polymer networks (IPNs); incorporation of more stable synthetic polymers (e.g., PEG and PVA); optimizing crosslinking density
Biocompatibility and tissue integration	Risk of inflammation, fibrosis, or poor adhesion to surrounding ocular tissues	Surface functionalization with bioactive peptides; incorporation of natural polymers (e.g., HA and collagen); anti-inflammatory modifications
Mechanical mismatch	Inability to fully replicate native vitreous viscoelasticity and support for retina	Fine-tuning crosslinking methods; using dual-crosslinking systems; tailoring polymer molecular weight and concentration
Controlled biodegradability	Premature or uncontrolled degradation affects long-term performance	Development of stimuli-responsive hydrogels; balancing enzymatic vs. hydrolytic degradability; hybrid natural-synthetic systems
Drug delivery limitations	Current hydrogels may have burst release or insufficient loading capacity	Incorporation of nanoparticles or liposomes; covalent drug–polymer conjugates; design of responsive hydrogels (pH, ROS, and enzymes)
Functionalization for tissue repair	Limited regenerative ability and lack of bioactivity	Incorporation of growth factors, stem-cell-supportive motifs, and ECM-mimetic peptides
